# Digital Endpoints for Assessing Instrumental Activities of Daily Living in Mild Cognitive Impairment: Systematic Review

**DOI:** 10.2196/45658

**Published:** 2023-07-25

**Authors:** Lauren Lawson, Ríona Mc Ardle, Sarah Wilson, Emily Beswick, Radin Karimi, Sarah P Slight

**Affiliations:** 1 School of Pharmacy Population Health Sciences Institute Newcastle University Newcastle Upon Tyne United Kingdom

**Keywords:** mild cognitive impairment, MCI, functional status, activities of daily living, instrumental activities of daily living, IADLs, digital technology, mobile phone

## Abstract

**Background:**

Subtle impairments in instrumental activities of daily living (IADLs) can be a key predictor of disease progression and are considered central to functional independence. Mild cognitive impairment (MCI) is a syndrome associated with significant changes in cognitive function and mild impairment in complex functional abilities. The early detection of functional decline through the identification of IADL impairments can aid early intervention strategies. Digital health technology is an objective method of capturing IADL-related behaviors. However, it is unclear how these IADL-related behaviors have been digitally assessed in the literature and what differences can be observed between MCI and normal aging.

**Objective:**

This review aimed to identify the digital methods and metrics used to assess IADL-related behaviors in people with MCI and report any statistically significant differences in digital endpoints between MCI and normal aging and how these digital endpoints change over time.

**Methods:**

A total of 16,099 articles were identified from 8 databases (CINAHL, Embase, MEDLINE, ProQuest, PsycINFO, PubMed, Web of Science, and Scopus), out of which 15 were included in this review. The included studies must have used continuous remote digital measures to assess IADL-related behaviors in adults characterized as having MCI by clinical diagnosis or assessment. This review was conducted in accordance with the PRISMA (Preferred Reporting Items for Systematic Reviews and Meta-Analyses) guidelines.

**Results:**

Ambient technology was the most commonly used digital method to assess IADL-related behaviors in the included studies (14/15, 93%), with passive infrared motion sensors (5/15, 33%) and contact sensors (5/15, 33%) being the most prevalent types of methods. Digital technologies were used to assess IADL-related behaviors across 5 domains: *activities outside of the home, everyday technology use, household and personal management, medication management,* and *orientation*. Other recognized domains—*culturally specific tasks* and *socialization and communication*—were not assessed. Of the 79 metrics recorded among 11 types of technologies, 65 (82%) were used only once. There were inconsistent findings around differences in digital IADL endpoints across the cognitive spectrum, with limited longitudinal assessment of how they changed over time.

**Conclusions:**

Despite the broad range of metrics and methods used to digitally assess IADL-related behaviors in people with MCI, several IADLs relevant to functional decline were not studied. Measuring multiple IADL-related digital endpoints could offer more value than the measurement of discrete IADL outcomes alone to observe functional decline. Key recommendations include the development of suitable core metrics relevant to IADL-related behaviors that are based on clinically meaningful outcomes to aid the standardization and further validation of digital technologies against existing IADL measures. Increased longitudinal monitoring is necessary to capture changes in digital IADL endpoints over time in people with MCI.

**Trial Registration:**

PROSPERO International Prospective Register of Systematic Reviews CRD42022326861; https://www.crd.york.ac.uk/prospero/display_record.php?RecordID=326861

## Introduction

### Background

Mild cognitive impairment (MCI) is a syndrome associated with significant changes in cognitive function and mild impairment in complex functional abilities [1]. MCI is prevalent in 6% of older adults aged ≥60 years worldwide, with the prevalence rising to 25% in adults aged between 80 and 84 years [2,3]. A diagnosis of MCI is associated with a 5-fold increased risk of developing dementia later on in life [4]. Dementia, a neurodegenerative disorder characterized by changes in cognitive functions and behaviors that severely interfere with daily activity and quality of life [5], is now recognized as a public health priority with currently available treatments unlikely to stop or reverse cognitive decline [5,6]. The early identification of MCI as a predementia phase is essential to understanding disease mechanisms and identifying novel drug targets. By drawing comparisons between people with MCI and normal aging, we may also be able to identify subtle markers indicating decline [7]. However, not all cases of MCI will progress to dementia, with only 8% to 15% receiving a dementia diagnosis within 5 years [8]. To improve the detection of MCI cases *at risk* of developing dementia, we must identify differential characteristics between those who remain cognitively and functionally stable over time and those who will develop dementia.

Impairment in the instrumental activities of daily living (IADLs) in people with MCI is a key predictor of progression to dementia and is considered central to functional independence [7]. IADLs refer to complex behaviors such as financial management, shopping, and medication use, which require higher-order cognitive function, whereas basic activities of daily living (BADLs) include activities essential to independent living, such as dressing, bathing, and continence [9]. There is limited guidance available for assessing IADL impairments in people with MCI; this has led to inconsistency in their identification [10]. The broader term *IADL-related behaviors* will be used throughout this review to refer to complex activities related to the ability to live independently in the community. IADL-related behaviors are most commonly measured using self-reported and informant-based questionnaires, which are susceptible to cultural, educational, gender, and recall biases [11,12]. Most validated IADL questionnaires do not consider everyday technology use as an activity; however, recommendations have been made to include this activity in future reiterations of tools, given the growing number of older adults using computers and smartphones [7,13]. Performance-based measures, which involve enactment of an IADL under observation, may provide an alternative to informant-based questionnaires; however, they usually require highly trained assessors, which may not be applicable or feasible in remote or marginalized communities [12]. They are also time consuming to collect and often take place in unnaturalistic settings that can bias functional performance [14]. The Manchester consensus on MCI recommends that changes in function, such as IADLs, be measured and monitored over time with technology in research and clinical practice [15].

The emergence of unobtrusive digital health technologies is a growing area of research to improve health care [16]. Devices such as ambient sensors and wearables allow us to objectively measure everyday behaviors in the real world and may provide a potential method to continuously assess IADL-related behaviors in people with MCI over time [17,18]; for example, the use of an electronic pillbox is a digital method that could be used to remotely monitor a person’s medication use. It could record metrics such as the time of day the pillbox was opened, a key IADL [19]. Wearable devices such as body-worn accelerometers have already been found to be a feasible method of measuring mobility (a BADL) in low- and middle-income countries [20]. A review of digital technologies for the monitoring of older adults found that a range of devices, including passive sensors and body-worn devices, were acceptable to users [21] and thus could be a promising method of assessment. Another review of nonwearable sensors found them able to overcome the limitations of traditional assessment tools and proposed that these devices could be useful supportive measures for the early detection and diagnosis of dementia [22].

The Early Detection of Neurodegenerative Diseases (EDoN) and Remote Assessment of Disease and Relapse–Alzheimer’s Disease (RADAR-AD) projects are 2 novel initiatives focused on the development of digital tool kits for the early detection of dementia. Both focus on using digital devices to measure clinically meaningful changes in individuals’ cognition and functional abilities over time. In the absence of disease-modifying treatments, early detection through the identification of subtle impairments is necessary to aid the development of preventive interventions [23]; therefore, it is important to understand which digital methods and metrics have been used to assess IADL-related behaviors in the literature and how these digital endpoints might differ between people with MCI and those aging normally and change over time. These findings may be useful for a multitude of different stakeholders, such as the pharmaceutical industry trialing the effectiveness of a new drug or the financial sector recognizing when an older adult might be losing their cognitive ability and become incapable of making informed decisions. By identifying the point of IADL change, interventions could be introduced to assist an individual through activities that they may no longer be able to complete (eg, cooking or financial management) independently [24]. This systematic review was conducted to address this knowledge gap.

### Aims

This review aimed (1) to identify the digital methods and metrics that have been used to assess IADL-related behaviors in people with MCI and (2) to report any significant differences in digital endpoints between people with MCI and normal aging and how these digital endpoints change over time.

## Methods

The review was preregistered with PROSPERO (CRD42022326861) and conducted in accordance with the PRISMA (Preferred Reporting Items for Systematic Reviews and Meta-Analyses) guidelines [25].

### Identification of Studies

#### Search Terms and Databases

Searches were conducted across 8 electronic databases, including CINAHL, Ovid (Embase, MEDLINE, and PsycINFO), ProQuest, PubMed, Scopus, and Web of Science. The results were restricted to articles published between January 1, 2004, and April 28, 2022, because 2004 is when MCI was first introduced as a clinical entity [26]. The full search strategy, including the combinations of terms used for each database, can be found in Multimedia Appendix 1.

#### Inclusion and Exclusion Criteria

The included studies must have used continuous remote digital measures to assess IADL-related behaviors in adults characterized as having MCI by clinical diagnosis or assessment. If the articles included cohorts other than adults with MCI, these cohorts were used for comparative purposes only. Peer-reviewed articles that were observational, cross-sectional, longitudinal, or interventional by design were included. Studies were excluded if their focus was on IADL measurement in populations with a clinical diagnosis of other health conditions, subjective cognitive impairment, or physical conditions that may have affected the participants’ cognition or function or if the studies were conducted in aged care facilities (ie, nursing homes and residential homes). Publications that used scripted tasks (ie, participants were given instructions to follow while being observed) or did not collect digital data were excluded. Studies that used domains of functional independence other than IADLs as an outcome measure (such as BADLs) were also excluded. Qualitative research, conference abstracts, case studies, literature reviews, and gray literature were also excluded. Finally, studies that were not published in English were excluded.

#### Selection Process

**Figure 1:**
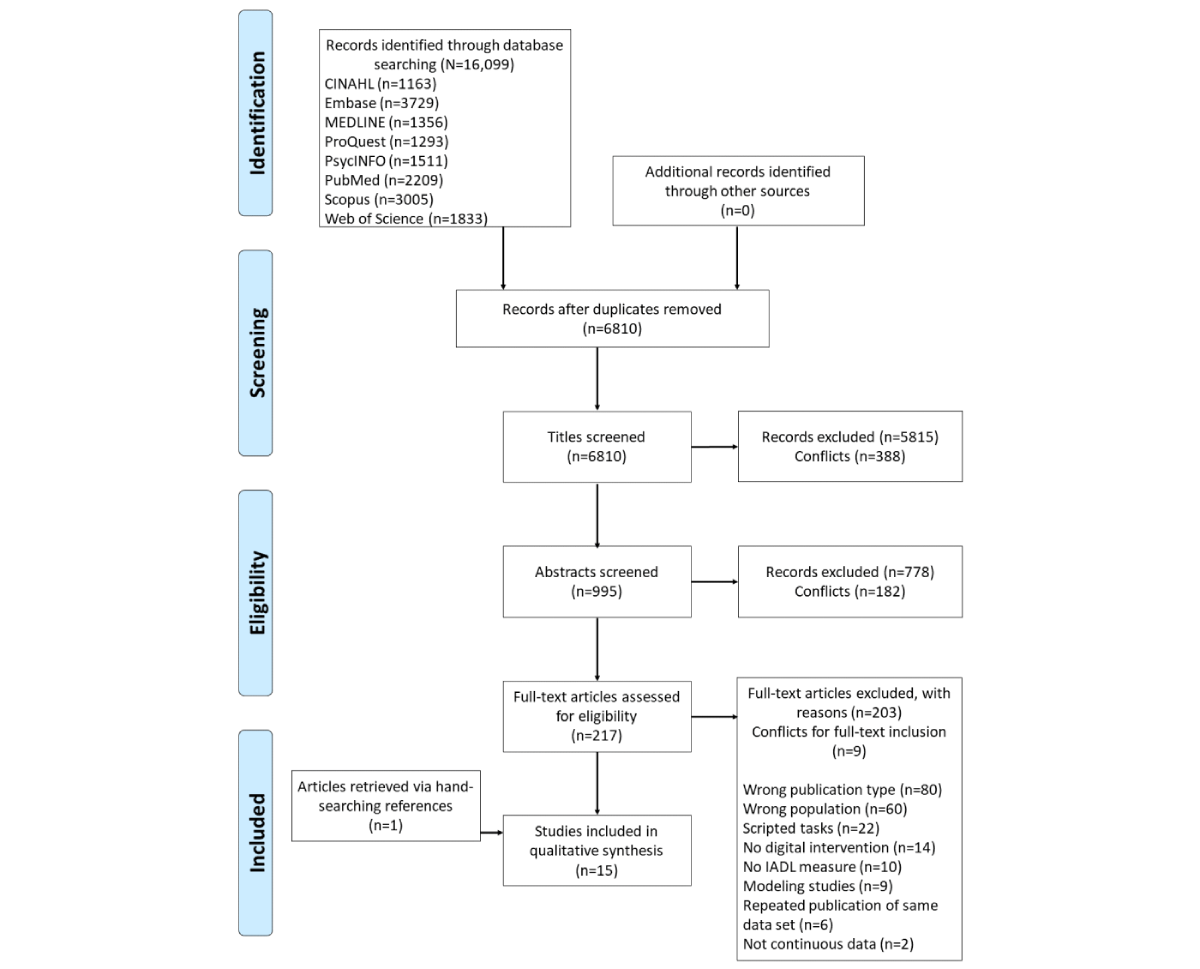
PRISMA (Preferred Reporting Items for Systematic Reviews and Meta-Analyses) diagram demonstrating the search yield for this review. IADL: instrumental activity of daily living.

All search results from each database were exported into referencing software (EndNote; Clarivate) and duplicates were removed. The remaining articles were then exported into Rayyan, an electronic software tool that we used to facilitate the blind screening of articles among 3 researchers (LL, SW, and RK) and the recording of reasons for article exclusion. Titles, abstracts, and full texts were reviewed against the set inclusion and exclusion criteria. A fourth, independent reviewer (RMA) resolved any disputes. The reference lists of the included studies were manually searched to identify any additional relevant studies that had not been detected in the search process (LL, SW, and RK; Figure 1).

### Data Analysis

#### Data Extraction

A data extraction sheet was developed to include the following information from the included studies: (1) details of the publication (authors, year of publication, and aims); (2) geographic location; (3) study design; (4) population (MCI, method of diagnosis, cognitive status, and method of cognitive assessment [eg, Mini-Mental State Examination; MMSE]); (5) sample size and demographic details; (6) traditional IADL assessment tools used; (7) type of technology (wearable, mobile app, or ambient); (8) location of technology (on body, in home, or portable); (9) validity, reliability, and acceptability of the technology; (10) period of data collection; (11) metrics pertaining to IADL-related behaviors; and (12) key findings relevant to the review aims.

#### Interpretation of Data

Jekel et al [7] and Yemm et al [12] have reported how there was limited standardization in the reporting of IADL impairments. Both reviews found different traditional scales to evaluate IADL-related behaviors, and thus, in this review, we synthesized the specific subdomains of IADLs from these papers into 7 broader categories: *activities outside of the home*, *culturally specific tasks*, *everyday technology use*, *household and personal management*, *medication management*, *orientation*, and *socialization and communication*. Two researchers (LL and EB) independently assessed each reported metric and included it in the most relevant domain.

#### Quality Assessment

Three reviewers (LL, SW, and RK) independently assessed the quality of each study, with a fourth reviewer (RMA) adjudicating in the case of disagreements. Quality assessment followed the National Institutes of Health Quality Assessment Tool for Observational Cohort and Cross-Sectional Studies [27]. This was adapted in line with the study by Mc Ardle et al [28] for specific application in reviews of populations with cognitive impairments and digital technology; evidence was rated as poor, mediocre, or good [28,29]. The results of the quality assessment can be found in Multimedia Appendix 2.

## Results

### Search Yield

The search identified 16,099 articles. After removing duplicates (n=9289), titles (n=6810) and abstracts (n=995) were screened, and 217 full texts were assessed for eligibility. In total, 14 articles met our inclusion criteria. We identified 1 additional article through manual searching of the reference lists of the included studies. Thus, 15 articles were included in this review (Figure 1). All studies were published between 2008 and 2022.

### Study Characteristics

The studies took place in the United States (11/15, 73%), the United Kingdom (1/15, 7%), Germany and Israel (1/15, 7%), Australia (1/15, 7%), and Singapore (1/15, 7%). The sample sizes of participant groups with MCI ranged from 7 to 76 individuals, with a mean age reported between 73 (SD 4.8) years and 88 (SD 11) years. The average years in education ranged from 4.5 to 16.3 (SD range 1.9-4.2) years. Of the 15 studies, 9 (60%) included ≥50% female participants. Ethnicity was reported in 7 (47%) of the 15 studies, with >70% of the study populations identifying as White. The study designs included longitudinal observational (11/15, 73%), cross-sectional feasibility (2/15, 13%), cross-sectional (1/15, 7%), and exploratory (1/15, 7%). Additional demographic information and study characteristics can be found in Table 1.

Cognitive assessments such as the MMSE or the clinical dementia rating (CDR) were used by 4 (27%) of the 15 papers to characterize MCI [30-33]. Of the 15 papers, 4 (27%) used validated diagnostic criteria such as the National Institute on Aging–Alzheimer’s Association workgroup criteria [1] to classify MCI [34-37], whereas 6 (40%) reported a consensus with ≥1 clinicians, alongside validated measures or cognitive assessment [38-43]. In 1 (7%) of the 15 papers, half of the sample with MCI was classified by cognitive assessment (CDR), whereas the remaining half was assessed by a clinician using validated criteria [44]. Comparison groups included cognitively intact older adult controls (13/15, 87%) [30-41,43,44], people with subjective cognitive decline (SCD; 1/15, 7%) [42], people with Alzheimer disease (1/15, 7%) [41], and people with unspecified dementia (1/15, 7%) [40].

**Table 1:** Participant demographic information for all included studies.

Study	Design	Country	Participant demographics	Diagnostic criteria
Bernstein et al [34]	Cross-sectional	United States	Participants with MCI^a^: n=21 Age (years): mean 75 (SD 6.2) Female participants: 23.8% Education (years): mean 14.8 (SD 3.3) Ethnicity: 95.2% White MoCA^b^ score: mean 22.7 (SD 2.3) FAQ^c^ score: mean 1.4 (SD 2.3) Controls: n=39 Age (years): mean 72.6 (SD 4.7) Female participants: 28.2% Education (years): mean 15.1 (SD 2.5) Ethnicity: 89.7% White MoCA score: mean 26.2 (SD 2.1) FAQ score: mean 0.6 (SD 1.6)	MCI: Albert et al [1]
Dodge et al [30]	Longitudinal observational	United States	Participants with incident MCI: n=11 Age (years): mean 86.0 (SD 6.2) Female participants: 79% Education (years): mean 14.6 (SD 1.9) Specific domains Category fluency (A+V^d^) score: mean 28.89 (SD 7.18) TMT-A^e^ score: mean 48.11 (SD 27.52) TMT-B^f^ score: mean 151.50 (SD 73.46) DST^g^ score: mean 35.32 (SD 12.06) LM-I^h^ score: mean 11.74 (SD 2.54) LM-D^i^ score: mean 10.05 (SD 2.84) BNT^j^ score: mean 25.42 (SD 3.15) Controls: n=86 Age (years): mean 84.2 (SD 4.9) Female participants: 85.8% Education (years): mean 15.5 (SD 2.4) Specific domains Category fluency (A+V) score: mean 31.31 (SD 7.17) TMT-A score: mean 41.80 (SD 17.10) TMT-B score: mean 120.81 (SD 57.45) DST score: mean 40.08 (SD 9.18) LM-I score: mean 13.67 (SD 4.05) LM-D score: mean 12.37 (SD 4.13) BNT score: mean 25.93 (SD 3.24)	MCI: Morris [45] (CDR^k^)
Dorociak et al [31]	Longitudinal observational	United States	Participants with incident MCI: n=9 Age (years): mean 84.4 (SD 7.1) Female participants: 89% Education (years): mean 16.3 (SD 2.0) Ethnicity: 89% White MMSE^l^ score: mean 28.4 (SD 1.3) FAQ score: mean 0.3 (SD 0.7) Controls: n=55 Age (years): mean 85.7 (SD 6.8) Female participants: 75% Education (years): mean 15.8 (SD 2.5) Ethnicity: 98% White MMSE score: mean 28.9 (SD 1.3) FAQ score: mean 0.3 (SD 0.7)	MCI: Morris [45] (CDR)
Hayes et al [32]	Cross-sectional	United States	Participants with MCI: n=7 Age (years): mean 88.4 Female participants: 57.1% Education (years): mean 14.4 MMSE score: mean 26.3 ADL^m^ score: mean 0.57 IADL^n^ score: mean 1.0 Controls: n=7 Age (years): mean 90 Female participants: 71.4% Education (years): mean 15.7 MMSE score: mean 27.9 ADL score: mean 0.14 IADL score: 0	MCI: Folstein et al [46] (MMSE) and Morris [45] (CDR)
Kaye et al [35]	Longitudinal observational	United States	Participants with MCI: n=38 Age (years): mean 83.4 (SD 4.8) Female participants: 84% Education (years): mean 15.5 (SD 2.2) Ethnicity: 76% White MMSE score: mean 28.5 (SD 1.4) FAQ score: mean 1.1 (SD 2.6) Controls: n=75 Age (years): mean 84.6 (SD 4.3) Female participants: 79% Education (years): mean 15.4 (SD 2.5) Ethnicity: 95% White MMSE score: mean 28.9 (SD 1.4) FAQ score: mean 0.5 (SD 1.3)	MCI: Petersen [26]
Leese et al [38]	Longitudinal observational	United States	Participants with MCI: n=18 Age (years): mean 75.4 (SD 7.7) Female participants: 50% Education (years): mean 15.2 (SD 3.5) Ethnicity: 83% White MoCA score: mean 23.06 (SD 2.13) FAQ score: mean 1.00 (SD 2.35) Controls: n=41 Age (years): mean 72.7 (SD 4.7) Female participants: 41.5% Education (years): mean 15.9 (SD 2.5) Ethnicity: 88% White MMSE score: mean 26.24 (SD 2.25) FAQ score: mean 0.07 (SD 0.26)	MCI: Albert et al [1] and consensus with clinician
Liddle et al [40]	Longitudinal observational	Australia	Participants with MCI: n=14; controls: n=3 Whole sample Age (years): mean 86.7 (SD 3.2) Female participants: 55.6% Education (years): mean 11.7 (SD 3.2)	MCI: Sachdev et al [47] and consensus with clinician
Petersen et al [33]	Longitudinal observational	United States	Participants with MCI: n=10; controls: n=75 Whole sample Age (years): mean 86.4 (SD 6.8) Female participants: 87.1% Ethnicity: 83.5% White	MCI: Morris [45] (CDR)
Rawtaer et al [39]	Cross-sectional feasibility	Singapore	Participants with MCI: n=28 Age (years): mean 75.1 (SD 6.3) Female participants: 67.9% Education (years): mean 4.5 (SD 3.9) MMSE score: mean 26.3 (SD 2.2) MoCA score: mean 24.0 (SD 2.2) Controls: n=21 Age (years): mean 73.0 (SD 5.3) Female participants: 66.7% Education (years): mean 7.0 (SD 4.0) MMSE score: mean 28.1 (SD 3.2) MoCA score: mean 27.5 (SD 1.6)	MCI: Petersen [26] and consensus with clinician
Seelye et al [43]	Longitudinal observational	United States	Participants with MCI: n=20 Age (years): mean 87.6 (SD 6.6) Female participants: 80% Education (years): mean 13.5 (SD 2.9) MMSE score: mean 27.3 (SD 1.4) Controls: n=42 Age (years): mean 87.9 (SD 5.2) Female participants: 88% Education (years): mean 15.6 (SD 2.5) MMSE score: mean 28.8 (SD 1.2)	MCI: Albert et al [1], Jak et al [48], and consensus with clinician
Seelye et al [37]	Longitudinal observational	United States	Participants with MCI: n=7 Age (years): mean 81.8 (SD 11.0) Female participants: 29% Education (years): mean 15.9 (SD 3.4) MMSE score: mean 28.9 (SD 1.6) FAQ score: mean 3.0 (SD 3.5) Controls: n=21 Age (years): mean 82.0 (SD 6.3) Female participants: 71% Education (years): mean 15.5 (SD 3.4) MMSE score: mean 28.8 (SD 1.5) FAQ score: mean 0.4 (SD 1.1)	MCI: Albert et al [1]
Seelye et al [36]	Cross-sectional feasibility	United States	Participants with MCI: n=15 Age (years): mean 74.3 (SD 6) Female participants: 6.7% Education (years): mean 14.9 (SD 2.3) Ethnicity: 100% White MoCA score: mean 22.9 (SD 1.8) FAQ score: mean 1.6 (SD 1.8) Controls: n=15 Age (years): mean 72.8 (SD 4.9) Female participants: 6.7% Education (years): mean 15 (SD 1.9) Ethnicity: 100% White MoCA score: mean 26.1 (SD 1.5) FAQ score: mean 0.4 (SD 1.1)	MCI: Morris [45] (CDR)
Stringer et al [42]	Proof-of-principle longitudinal	United Kingdom	Participants with MCI: n=14 Age (years): mean 74.3 (SD 4.8) Female participants: 42.9% Education (years): mean 12.9 (SD 3.2) ACE-III^o^ score: mean 88.36 (SD 4.73) Participants with SCD^p^: n=18 Age (years): mean 71.1 (SD 3.4) Female participants: 72.2% Education (years): mean 13.4 (SD 3.5) ACE-III score: mean 96.28 (SD 3.49)	MCI: Petersen [26], clinical diagnosis, and consensus with clinician; and SCD: Farias et al [49] (ECog^q^)
Wettstein et al [41]	Longitudinal observational	Germany and Israel	Participants with MCI: n=76 Age (years): mean 72.9 (SD 6.5) Female participants: 51.3% Education (years): mean 12.3 (SD 4.2) MMSE score: mean 27.0 (SD 2.1) SF-36^r^ score: mean 77.2 (SD 21.8) Participants with AD^s^: n=35 Age (years): mean 74.1 (SD 7.1) Female participants: 40% Education (years): mean 12.5 (SD 3.2) MMSE score: mean 24.1 (SD 2.4) SF-36 score: mean 78.7 (SD 24.8) Controls: n=146 Age (years): mean 72.5 (SD 6.1) Female participants: 50% Education (years): mean 14.5 (SD 4.2) MMSE score: mean 27.5 (SD 2.3) SF-36 score: mean 80.5 (SD 19.7)	MCI: Winblad et al [50] and consensus with clinician, and AD: Levy [51]
Wu et al [44]	Longitudinal observational	United States	Participants with MCI: n=19 Age (years): mean 73.1 (SD 7.5) Female participants: 47.4% Education (years): mean 14.9 (SD 3.4) Ethnicity: 89.5% White Controls: n=120 Age (years): mean 78.9 (SD 8.5) Female participants: 78.3% Education (years): mean 15.8 (SD 2.7) Ethnicity: 75% White	MCI: half using Morris [45] (CDR) and half by clinician using validated criteria

^a^MCI: mild cognitive impairment.

^b^MoCA: Montreal cognitive assessment.

^c^FAQ: Functional Activities Questionnaire.

^d^A+V: animals+vegetables.

^e^TMT-A: Trail Making Test A.

^f^TMT-B: Trail Making Test B.

^g^DST: Digit Symbol Test.

^h^LM-I: Logical Memory Test (Immediate Recall).

^i^LM-D: Logical Memory Test (Delayed Recall).

^j^BNT: Boston Naming Test.

^k^CDR: clinical dementia rating.

^l^MMSE: Mini-Mental State Examination.

^m^ADL: activity of daily living.

^n^IADL: instrumental activity of daily living.

^o^ACE-III: Addenbrooke Cognitive Examination-III.

^p^SCD: subjective cognitive decline.

^q^ECog: everyday cognition.

^r^SF-36: 36-item Short-Form Health Survey.

^s^AD: Alzheimer disease.

### Digital Methods to Measure IADLs

Only 5 (71%) of the 7 aforementioned IADL domains were found to have been digitally assessed across the studies. These included *activities outside of the home* (8/15, 53%) [32,33,37-41,44], *everyday technology use* (7/15, 47%) [30,34-36,38,42,43], *medication management* (3/15, 20%) [31,36,39], *household and personal management* (1/15, 7%) [39], and *orientation* (2/15, 13%) [40,44]. No studies digitally assessed behaviors related to *socialization and communication* or *culturally specific tasks*.

**Figure 2:**
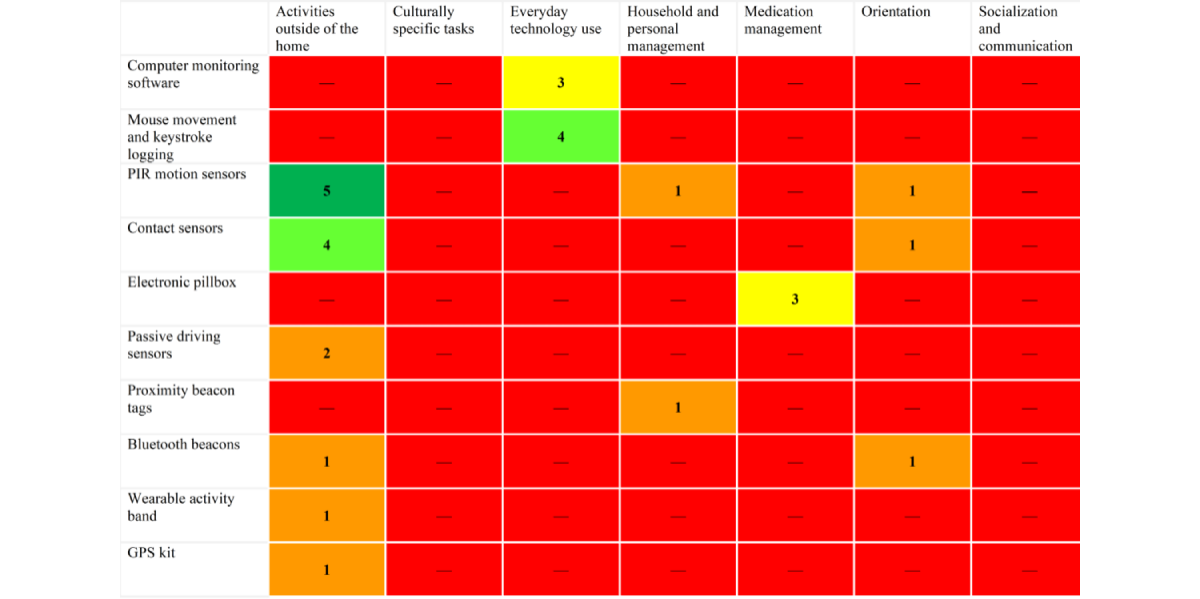
Heat map showing the number of technologies and the instrumental activity of daily living domain they measured. PIR: passive infrared.

Ambient technology in the home was the most popular method of assessment, with 93% (14/15) of the studies measuring IADLs using this method [30-40,42-44]. The types of ambient technologies that were used included passive infrared (PIR) motion sensors (5/15, 33%) [32,33,35,39,44], contact sensors (5/15, 33%) [32,33,35,39,44], mouse movement and keystroke logging (4/15, 27%) [30,35,42,43], computer monitoring software (3/15, 20%) [34,36,38], electronic pillboxes (3/15, 20%) [31,36,39], passive driving sensors (2/15, 13%) [37,38], proximity beacon tags (1/15, 7%) [39], and Bluetooth beacons (1/15, 7%) [40]. PIR motion sensors were usually placed in each room to monitor activity throughout the home and routinely coupled with contact sensors placed in doorways to measure the opening and closing of doors; these sensors monitored the amount of time participants spent at home in the *activities outside of the home* and *orientation* domains. Proximity beacon tags were attached to personal items such as keys or wallets to monitor how often they were left in the home when the participants were logged as out of home with other sensors in the *household and personal management* domain. The other technologies used are defined in Multimedia Appendix 3. Only 3 (20%) of the 15 studies used wearable technology in the form of wearable activity bands (n=1, 33%) [39], wearable smartphones (n=1, 33%) [40], and a portable GPS kit (n=1, 33%) [41]. The wearable smartphones used a GPS app to measure time spent and distance traveled outside of the home [40] and, in another study, smartphones were used ambiently to process driving data (1/15, 7%) [37]. The details of all different technology types and IADL domains measured can be found in Figure 2.

The data collection period varied among the studies, ranging from 7 to 1095 days, with 7 days being the most common study duration (3/15, 20%) [30,40,43]. Of the 15 studies, 8 (53%) reported data loss during the monitoring period owing to technical or logistic problems [32,33,36-39,41]. Only 1 (7%) of the 15 studies reported information on the sensitivity (94%) and specificity (98%) of their in-home sensor platform for assessing time out-of-home compared with motion-activated video cameras [33]. None of the included studies compared their digital technologies against existing validated measures of IADL assessment, such as the Lawton and Brody Scale [52]. Only 2 (13%) of the 15 studies reported on participants’ acceptability of the devices [37,39]. Of these 2 studies, 1 (50%) reported that 83% of the participants provided positive feedback on the multiple monitoring devices used (PIR motion sensors, contact sensors, electronic pillboxes, proximity beacon tags, and wearable activity bands) and felt secure and safe when using the motion and contact sensors [39], and 1 (50%) reported that 89% of the participants found the remote driving sensors and smartphones located in their vehicles acceptable [37]. Information relating to the key results of each study can be found in Multimedia Appendix 3.

### Digital Metrics to Assess IADL-Related Behaviors

A total of 79 different metrics were reported across 5 domains in the included studies. All metrics are described, and their frequency reported, in Multimedia Appendix 4. Of the 15 studies, 7 (47%) assessed *everyday technology use* by measuring 35 discrete metrics [30,34-36,38,42,43]. Only 1 (14%) of these 7 studies recorded metrics for the time spent on individual computer applications, including emailing, gaming, teleconferencing, finance use, and search use [34]. The same study also recorded the percentage of days with at least 1 computer session [34], whereas another study calculated the average number of days spent using the computer per month [35]. Of these 7 papers, 1 (14%) reported 10 discrete mouse movement metrics, including the straight-line distance, total distance, time taken (milliseconds) to make each movement, curvature, and time (milliseconds) spent idling or pausing, alongside their ranges, among the participants [43]. The same study also recorded the total number of mouse movements as well as the total number of computer sessions [43], whereas another study measured the number of mouse clicks per day and daily keystroke speed [42].

Of the 15 studies, 8 (53%) assessed *activities outside of the home* by measuring 31 discrete metrics. Time out-of-home was the most common metric, measured by 6 (75%) of the 8 studies and reported by 4 (50%) of the 8 studies, with mean scores ranging from 62 to 258 (SD range 70.9-142.3) minutes per day [32,33,39,41]. The mean number of outings per day ranged from 0.96 to 1.00 in 2 (50%) of the 4 studies [32,39]. A daily activity estimate was used to measure time out-of-home in 2 (25%) of the 8 studies [32,35]. Two other metrics related to time—the percentage of time spent at home and the number of days in the week on which participants left home—were reported by 1 (7%) of the 15 studies [40]. Of the 8 studies, 1 (13%) reported the furthest distance (kilometers) traveled from the home [40], whereas another reported the number of places visited [41]. Of the 8 studies, 2 (25%) reported the average time spent driving per day, with a range of 48 to 65.2 minutes recorded [37,38]. The same 2 studies also reported the number of daily trips in a vehicle and the time (seconds) spent driving on highways and at night [37,38]. Other driving metrics that were also recorded include the number of hard brakes, hard accelerations, turns (in either direction), and time (seconds) spent driving at speeds of >70 mph [37]. This study also recorded the percentage of days when participants drove ≥20 miles as well as the total number of days during which their driving was monitored [37], whereas another study recorded the furthest distance (meters) traveled per day [38].

Four metrics were measured in the *medication management* domain by 3 (20%) of the 15 studies [31,36,39]. The time of day the pillbox was opened (pill-taking clock time) was measured by 2 (67%) of the 3 studies [31,36]; of these 2 studies, 1 (50%) recorded variability in the time of day the pillbox was opened [31]. Another study reported the mean frequency of forgetting medication, derived from baseline medication information and the times the pillbox was opened, as 30 (SD 28) times per month [39]. Six metrics were used to measure *orientation* in 2 (13%) of the 15 studies [40,44]. The number of room-to-room transitions made in a day and the variance of these transitions within 1 week were reported in 1 (50%) of these 2 studies [44]. Metrics regarding the geographic area traveled by a participant, known as lifespace, were collected by another study [40]. Only 1 (7%) of the 15 studies measured IADL-related behaviors in the *household and personal management* domain, using 2 metrics: the frequency of forgetting keys and the frequency of forgetting a wallet [39].

### Comparisons in Digital Endpoints Between People With MCI and Normal Aging, and How These Change Over Time

#### Overview

All 15 studies explored differences in IADL digital endpoints between people with MCI and other cognitive groups. Of the 15 studies, 5 (33%) investigated how IADL digital endpoints changed over time in people with MCI. Table 2 summarizes the variations in key cross-sectional and longitudinal findings for each metric reported by the included studies.

**Table 2:** Correlation table showing cross-sectional and longitudinal key findings for cognitive groups compared with people with mild cognitive impairment.^a^

Domain and metric		Total number of studies	Cognitive groups			
			Cognitively intact	Subjective cognitive decline	Alzheimer disease	Dementia
**Activities outside of the home**						
	Time out-of-home	4	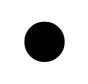 ^b^ 3^c^; 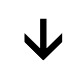 ^d^ 1	—^e^	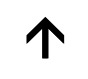 ^f^ 1	—
	Daily distance (meters)	1	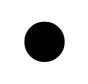 1	—	—	—
	Number of trips/day	2	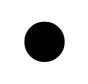 2	—	—	—
	Day-to-day variability in number of trips	1	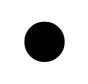 1	—	—	—
	Distance driven/day (miles)	1	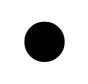 1; 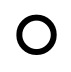 ^g^ 1	—	—	—
	Day-to-day variability in distance driven	1	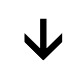 1	—	—	—
	Time driven/day (hours and min)	2	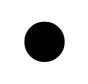 2; 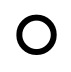 1	—	—	—
	Day-to-day variability in time driven	1	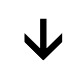 1	—	—	—
	First clock start time	1	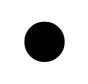 1	—	—	—
	Day-to-day variability in first start time (hours)	1	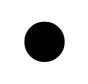 1	—	—	—
	Last clock start time	1	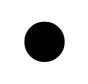 1	—	—	—
	Day-to-day variability in last start time (hours)	1	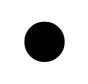 1	—	—	—
	Number of days monitored	1	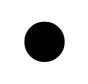 1	—	—	—
	Percentage of days at least 1 trip was taken out of all days monitored	1	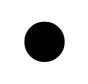 1	—	—	—
	Percentage of driving days with ≥20 miles driven	1	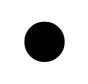 1	—	—	—
	Highway driving/day (seconds)	2	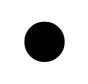 1; 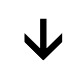 1	—	—	—
	Nighttime driving/day (seconds)	2	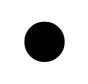 2	—	—	—
	Left turns/day	1	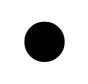 1	—	—	—
	Right turns/day	1	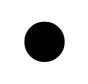 1	—	—	—
	Time driving at speeds of >70 mph/day	1	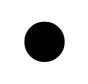 1	—	—	—
	Hard brakes/day	1	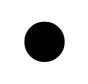 1	—	—	—
	Hard accelerations/day	1	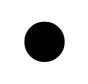 1	—	—	—
	Number of outings/day	2	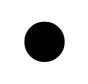 2	—	—	—
	Nodes (places) visited/day	1	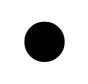 1	—	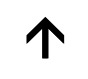 1	—
	Trips away from home/week	1	—	—	—	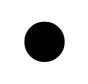 1
	Daily activity estimate	1	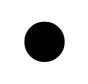 1	—	—	—
	CoV^h^ of activity	1	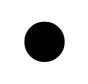 1	—	—	—
	24-hour wavelet analysis of activity variance	1	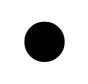 1	—	—	—
	Percentage of time at home	1	—	—	—	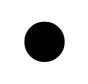 1
	Average maximum distance from home	1	—	—	—	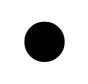 1
	Days in the week when participant left home	1	—	—	—	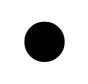 1
**Everyday technology use**						
	Computer use time	5	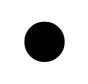 3; 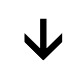 1; 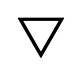 ^i^ 2	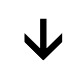 1; 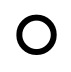 1	—	—
	Computer use time variability	1	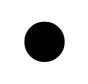 1	—	—	—
	Number of sessions	1	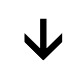 1	—	—	—
	Time of first session	1	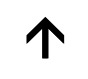 1	—	—	—
	Time of last session	1	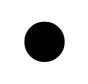 1	—	—	—
	Percentage of days with at least 1 session	1	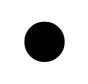 1	—	—	—
	Email use time (min)	1	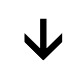 1	—	—	—
	Email use time (days)	1	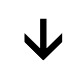 1	—	—	—
	Browser use time (min)	1	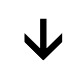 1	—	—	—
	Browser use time (days)	1	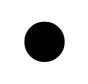 1	—	—	—
	Search use (min)	1	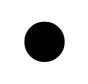 1	—	—	—
	Search use (days)	1	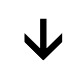 1	—	—	—
	Word processing use time (min)	1	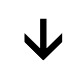 1	—	—	—
	Word processing use (days)	1	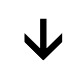 1	—	—	—
	Game use time (min)	1	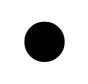 1	—	—	—
	Game use time (days)	1	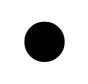 1	—	—	—
	Teleconferencing use time (min)	1	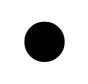 1	—	—	—
	Teleconferencing use time (days)	1	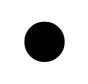 1	—	—	—
	Finance use time (min)	1	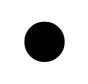 1	—	—	—
	Finance use time (days)	1	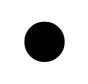 1	—	—	—
	Days with computer use	1	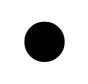 1; 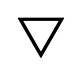 1	—	—	—
	CoV of use	1	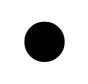 1; 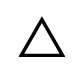 ^j^ 1	—	—	—
	Median delta	1	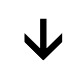 1	—	—	—
	IQR delta	1	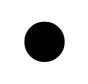 1	—	—	—
	Median D	1	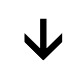 1	—	—	—
	IQR D	1	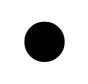 1	—	—	—
	Median T	1	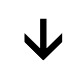 1	—	—	—
	IQR T	1	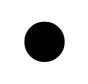 1	—	—	—
	Median K	1	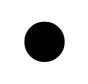 1	—	—	—
	IQR K	1	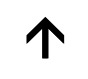 1	—	—	—
	Median idle	1	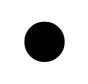 1	—	—	—
	IQR idle	1	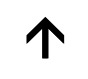 1	—	—	—
	Number of mouse movements contributed	1	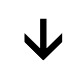 1	—	—	—
	Total number of sessions	1	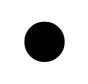 1	—	—	—
	Keystroke speed	1	—	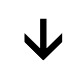 1; 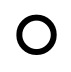 1	—	—
	Mouse click frequency	1	—	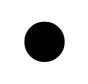 1; 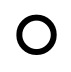 1	—	—
**Medication management**						
	Medication adherence	1	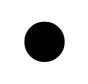 1; 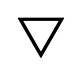 1	—	—	—
	Pill-taking clock time	1	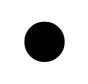 1; 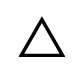 1	—	—	—
	Pill-taking clock time variability	1	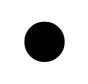 1; 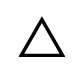 1	—	—	—
	Frequency of forgetting medication/month	1	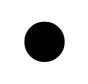 1	—	—	—
**Household and personal management**						
	Frequency of forgetting keys/month	1	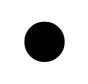 1	—	—	—
	Frequency of forgetting wallet/month	1	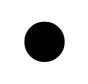 1	—	—	—
**Orientation**						
	Indoor mobility frequency	1	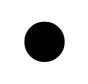 1	—	—	—
	Indoor mobility stability	1	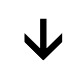 1	—	—	—
	Daily lifespace area	1	—	—	—	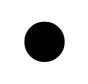 1
	Total lifespace area	1	—	—	—	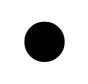 1
	Indoor lifespace	1	—	—	—	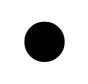 1
	Lifespace score	1	—	—	—	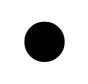 1

^a^Studies may have reported >1 result.

^b^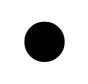: no significant results.

^c^Number refers to the number of included studies reporting a result.

^d^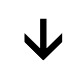: significant decrease.

^e^Not available.

^f^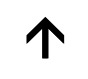: significant increase.

^g^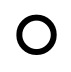: no significant change over time.

^h^CoV: coefficient of variation.

^i^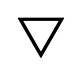: significant decrease over time.

^j^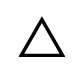: significant increase over time.

#### Activities Outside of the Home

Of the 15 studies, 7 (47%) looked at comparisons between people with MCI and controls for metrics related to activities outside of the home. Of these 7 studies, 1 (14%) looked at comparisons between people with MCI and those with Alzheimer disease as well as controls [41], and another looked at comparisons between people with MCI and those with unspecified dementia [40]. Of the 7 studies, 1 (14%) assessed longitudinal changes [37].

In comparison with normal aging, most of the studies (4/6, 67%) found no differences in IADL metrics of activities outside of the home between people with MCI and controls [38-41]. Of the 6 studies, 3 (50%) found no differences in time out-of-home between people with MCI and controls [32,39,41], although 1 (33%) of these 3 studies reported a trend toward people with MCI spending less time out-of-home, but these results were not statistically significant [32]. Another study reported that people with MCI spent an average of 1.67 hours more inside the home than controls and were 12% less likely to leave the home at all on any given day [33]. Of the 6 studies, 1 (17%) also reported no differences in the number of places visited between people with MCI and controls [41]. Overall, 2 (33%) of the 6 studies found no differences in the number of outings between people with MCI and controls [32,39], with a small nonsignificant trend toward people with MCI having fewer outings per day recorded in 1 (50%) of the 2 studies [32]. Greater variation in the day-to-day pattern of activity was reported for people with MCI in 1 (17%) of the 6 studies [32]. There were inconclusive findings for differences in driving behaviors, with 1 (17%) of the 6 studies reporting that the group with MCI spent less time driving per day than controls, drove for fewer miles, and spent less time driving on highways [37]; however, another study reported no differences in these metrics between people with MCI and controls [38]. Findings were inconclusive for comparisons of *activities outside of the home* metrics between people with MCI and those with other neurodegenerative conditions. One study reported no differences in the time out-of-home, maximum distance from home, the number of outings, and days when participants left home between people with MCI and those with dementia [40]. However, another study reported that people with Alzheimer disease visited fewer places than those with MCI and spent less time out-of-home [41].

#### Changes in Activities Outside of the Home Over Time

Only 1 (14%) of the 7 studies investigated changes over time in the driving metrics, total daily distance, and total daily driving time and found no differences between the group with MCI and controls [37].

#### Everyday Technology Use

Of the 15 studies, 6 (40%) compared *everyday technology use* metrics between people with MCI and controls. Of these 6 studies, 1 (17%) looked at comparisons between people with MCI and those with SCD [42], and 3 (50%) looked at longitudinal changes in these metrics [30,33,42].

When comparing people with MCI to normal aging, 3 (50%) of the 6 studies found no differences between people with MCI and controls for daily computer use [30,35,38]; however, 1 (17%) of the 6 studies reported that people with MCI spent less time using the computer than controls, and they also had fewer sessions, a later first use time, and less variability in their use time [34]. The same study also found that the group with MCI spent fewer minutes using email, web browsers, and word processing, as well as fewer total days using search tools and word processing. Of the 6 studies, 1 (17%) found no differences for days with computer use between people with MCI and controls [35]. Another study found that people with MCI made shorter mouse movements than controls, and they also took less time for each movement, made larger and more variable curved or looped movements, had larger and more variable pauses between movements, and had fewer total movements [43]. Another study found that the group with MCI spent less time on the computer and had a slower keystroke speed than people with SCD [42].

#### Changes in Everyday Technology Use Over Time

Of the 6 studies, 1 (17%) found that over an average of 36 months, people with MCI had a decrease of approximately 1% per month in their mean daily use of the computer, a decrease in the number of days they used the computer, and an increase in day-to-day use variability compared with controls [35]. Another study reported that controls had less decline over time in weekly average minutes on the computer than people with incident MCI (those who developed MCI during the monitoring period) and that, over time, participants in the group with incident MCI spent fewer minutes on their computer [30]. Another study found no significant changes over time in daily computer use, mouse click frequency, or keystroke speed in individuals with MCI or SCD [42].

#### Medication Management

Of the 15 studies, 3 (20%) assessed *medication management*; however, only 2 (67%) of these 3 studies compared people with MCI and controls and reported these results [31,39]. Of these 2 studies, 1 (50%) assessed longitudinal changes in this domain [31]. In comparison with people aging normally, 2 (67%) of the 3 studies found no significant differences in medication-taking metrics between people with MCI and controls [31,39].

#### Changes in Medication Management Over Time

The only study to assess changes in *medication management* over time reported that the group with incident MCI opened their electronic pillboxes increasingly later in the day—by 19 minutes per month—than controls and became more variable in terms of the first time they opened the pillbox each day [31]. The same study also reported how medication adherence significantly decreased in both groups over the 2-year study period.

#### Orientation

*Orientation* was assessed by 2 (13%) of the 15 studies cross-sectionally, with no longitudinal findings [40,44]. Of these 2 studies, 1 (50%) compared people with MCI and controls, whereas the other compared people with MCI and those with unspecified dementia. The study comparing people with MCI and normal aging found the group with MCI to have lower indoor mobility stability, indicating a higher day-to-day variability per week than controls [44]. The study comparing people with MCI and those with unspecified dementia found no differences in indoor lifespace, average lifespace, and total lifespace, as well as in lifespace scores [40].

#### Household and Personal Management

Only 1 (7%) of the 15 studies assessed *household and personal management* cross-sectionally, with no longitudinal data. No significant differences between people with MCI and controls were reported [39].

## Discussion

### Overview

**Figure 3:**
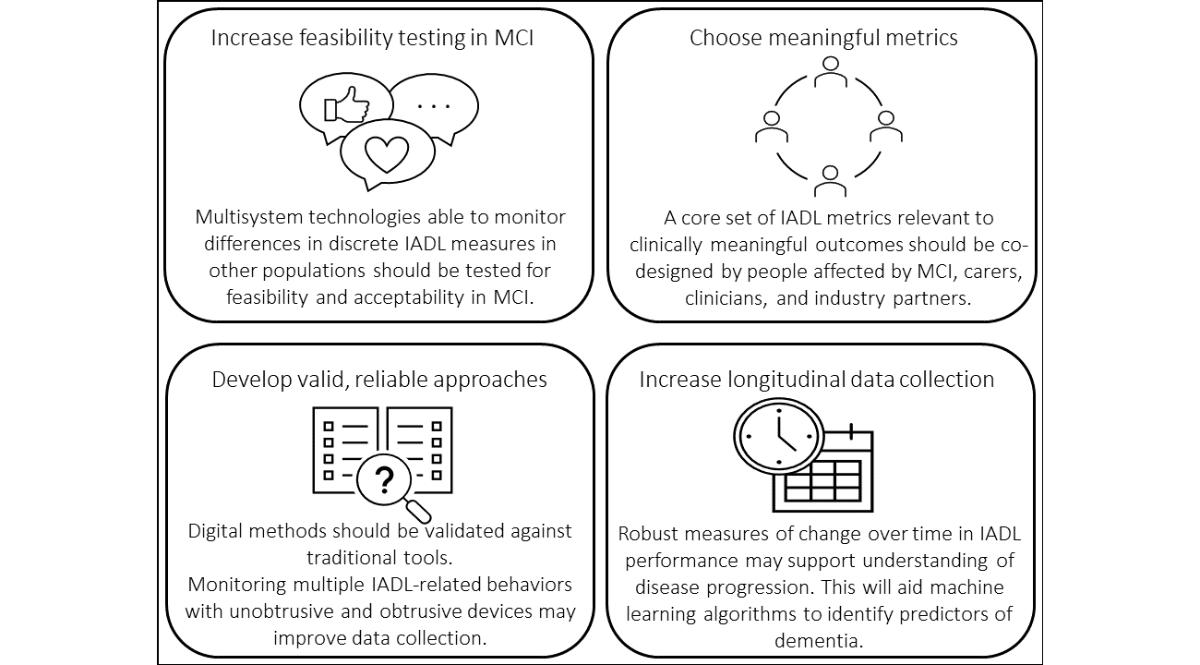
Recommendations for future research regarding the assessment of instrumental activities of daily living (IADLs)–related digital endpoints in people with mild cognitive impairment (MCI).

This systematic review is the first to report the different digital methods and metrics used to assess IADL-related behaviors in people with MCI and explore significant differences in IADL-related digital endpoints between people with MCI and normal aging, and how these digital endpoints change over time. Only 5 (71%) of the 7 IADL domains were found to have been digitally assessed across the studies, and 65 (82%) of the 79 metrics reported in this review were only used once (Multimedia Appendix 4). The use of ambient technology, such as PIR motion sensors and contact sensors, was found to be the most prevalent *method*, whereas computer use time was one of the most common *metrics* related to *everyday technology use*. There were inconsistent findings regarding differences in digital IADL endpoints across the cognitive spectrum and limited longitudinal assessment of how they changed over time. On the basis of these findings and their implications, key considerations for the measurement of IADLs in people with MCI using digital endpoints have been highlighted and summarized in Figure 3.

### Digital Methods and Metrics for Measuring IADLs in People With MCI

Most of the studies (14/15, 93%) used ambient technology to digitally assess IADL-related behaviors. This is somewhat consistent with the literature because ambient *smart home* systems have been used in multiple cohorts to measure IADLs [53,54]; for example, the Sensor Platform for Healthcare in a Residential Environment (SPHERE) system, which has been used to monitor IADL-related behaviors in people with Parkinson disease, comprises a wrist-worn accelerometer alongside multiple environmental sensors (including motion sensors similar to those found in this review) that can also assess energy consumption, humidity, and temperature [55]. This system has been extended to groups with cognitive impairment in current pilot work with people with MCI and Alzheimer disease [56]. Other research has collected more discrete targeted IADL-related behaviors; for example, analog sensors that measure energy consumption have been used to capture cooking activities such as stove burner use [57]. A *smart plug* that monitors energy consumption was used by 1 (7%) of the 15 included studies to capture television use, but this technology was not used to measure IADLs in people with MCI [39]. This research area is still in its infancy, with huge scope to expand existing methods for different disease states.

Financial management is one of the first IADLs to deteriorate in the early stages of dementia and experience impairment in people with MCI [58,59]; however, this was only measured by 1 (7%) of the 15 included studies under computer use [34]. Developing metrics around the use of electronic banking has potential, although we need to be mindful that half of the adults aged between 65 and 74 years do not actually use this facility [60], which may currently be a limiting factor. Although this review was focused on continuous technology, noncontinuous technology has been used to assess this IADL, such as scripted banking tasks available on smartphones [61]. There was no standardization of metrics in all IADL domains measured [30-44]. There was also no standardization of metrics for studies measuring IADL-related behaviors with the same technology [37,38]. All 36 metrics reported in the domain *everyday technology use* related to home computer use; no other technology was assessed. By contrast, validated pen-and-paper measures in the literature have included items assessing the management of electronic household appliances, remote controls, and mobile phones [62-64]; this suggests that there are several potentially relevant metrics that are not being collected digitally.

A core set of IADL-related digital endpoints should be developed in partnership with people with MCI, carers, clinicians, and industry partners, whereby the digital endpoints are clinically meaningful and relevant to patient-related outcomes (eg, independence and quality of life) and useful to capture in this population [7,12]. This would support the standardization of IADL-related digital endpoints and allow a more comprehensive understanding of how these digital endpoints might be useful for identifying MCI and monitoring disease progression.

### Digital Endpoints in MCI: Comparisons Across the Cognitive Spectrum and Changes Over Time

This review found no strong evidence of differences in IADL digital endpoints between MCI and normal aging or dementia. This contradicts the findings of previous studies, where significant deficits in IADL-related behaviors in patients with MCI were reported [7]. A meta-analysis by Lindbergh et al [65] found that the performance of IADLs is impaired in people with MCI at baseline, with the largest differences in IADLs recorded by performance- and questionnaire-based assessments in comparison with self-report. This meta-analysis contained studies that used traditional measures, such as performance- and questionnaire-based assessments, that may be subject to cultural, educational, gender, and recall biases [11,12] and be collected in unnaturalistic settings that can bias functional performance [14]. Our review only included studies that used continuous remote digital measures to assess IADL-related behaviors. In the literature, unobtrusive in-home sensors have been used to continuously measure multiple IADL-related behaviors, such as cooking and socializing, alongside several BADLs to establish differences in routine behaviors between people with dementia and controls [66]; researchers found increased variability in the performance of these activities in people with dementia compared with controls [66]. This suggests that measuring multiple IADL-related behaviors at the same time using multisystem digital technology can detect differences in routine across different levels of cognitive impairment, which is useful for understanding the timing of changes indicative of further functional decline; for example, changes in routine such as leaving a stove on can be an indicator for greater care provision, such as the transition into care facilities [67]. However, this review also suggests that measuring multiple IADL-related digital endpoints may be more useful than measuring discrete IADL outcomes alone to observe changes in function and inform machine learning algorithms focused on detecting changes over time [68].

There was limited longitudinal research identified in this review, highlighting a gap in our understanding of how the digital assessments of IADL-related behaviors might detect change over time relevant to cognitive decline and disease progression. Only 2 (13%) of the 15 studies reported longitudinal changes in IADL-related behaviors, reporting increased variability in medication management and computer use in people with MCI over a monitoring period ranging from 28 to 36 months [31,35]. This is somewhat consistent with previous studies, whereby people with MCI identified medication management as one of the earliest IADLs that they are unable to perform independently [69]. As previously mentioned, increased variability in daily behavioral patterns has been documented in people with dementia [66]; therefore, increasing variability in the performance of IADLs may be a suitable digital endpoint for monitoring the progression of MCI. IADL-related behaviors were not explored as digital endpoints for interventions but could be considered in the future with growing evidence of their utility in other clinical populations [70]; for example, an integrated system of wrist-worn and ambient sensing has been deployed for postoperative monitoring of people after hip and knee replacement surgery and heart valve intervention to monitor recovery [70,71]. Quantitative validation against patient-reported outcome measures is ongoing; however, participants using the system have reported positive feedback, which suggests that this could also be useful to monitor treatment or intervention effects in people with MCI [72].

### Limitations of This Research and Key Considerations

A key limitation in the included studies was the lack of information on the validity of the digital technology used to assess IADL-related behaviors, with only 1 (7%) of the 15 studies reporting the sensitivity and specificity of the digital technology used [33]. Concurrent validity among the digital measures to assess IADL-related behaviors and established IADL performance- and questionnaire-based assessments was not reported by any study. This is a consistent issue in the literature; a review of technologies used to measure BADLs and IADL-related behaviors in people with Parkinson disease also found a lack of reported validation for measures of both, highlighting this to be a relatively unexplored area of research [73]. Therefore, it is currently unclear how accurately these devices are measuring IADL-related behaviors or how effective they might be to detect changes in IADL-related behaviors over time. Future research in this area should ensure that digital technologies used to assess IADL-related behaviors are valid, reliable, and accurate and report or cite validity information where available. Digital technologies should also be validated against current scales used to assess IADLs to prove that they are measuring similar constructs.

This review found that only 2 (13%) of the 15 studies provided information on the acceptability of these digital technologies to users [37,39], and 1 (7%) of the 15 studies found that participants with MCI required more technology maintenance visits than the control group [36]; this suggests that people with MCI may struggle to remotely troubleshoot their devices, which is an important consideration when developing technology for use in a population with cognitive impairment. Participation in several of the studies (6/15, 40%) was limited to single-occupant households because the monitoring technology used was unable to differentiate among people living in the same household. This is an important finding; proximity tags could be used in multiperson households to identify the person who is being monitored; however, this would mean that the technology would no longer be unobtrusive [32]. Considering the amount of data digital technology can collect, attitudes toward data privacy were not addressed by the included studies. Qualitative research investigating participants’ privacy concerns highlighted motivation, transparency, and trust to be among the key themes that influence how they felt about their data being used [74]. With this in mind, future work should increase feasibility and acceptability testing of digital technology in people with MCI to maintain transparency and trust between researchers and participants.

A significant limitation highlighted in this review is the inconsistent classification of MCI across the included studies (eg, clinician consensus, use of diagnostic criteria, and cognitive score thresholds). Diagnosing MCI has been highlighted as inconsistent, with widespread variation in the rates of diagnosis among services [15]. This limits the extent to which the sample represents MCI, which in turn may explain the inconsistency in findings. Consistent, accurate diagnosis of MCI through the use of clinical consensus is needed to observe clinically meaningful changes in IADLs. In addition, information regarding the inclusivity and representativeness of the sample populations in the reviewed studies was limited; for example, more than half of the papers (8/15, 53%) did not report ethnicity [30,32,37,39-43], which limits our understanding of how IADL-related behaviors may be assessed digitally in a representative group of people with MCI.

### Implications

Digital technologies are rapidly progressing in the field of neurodegenerative diseases [21]. They can collect continuous objective real-life patient data that can indicate cognitive decline, which has many applications both clinically and in the performance of research studies [21]. Subtle changes in activities of daily living detected by these technologies can be used to assist diagnosis; for example, gait characteristics identified with wearable accelerometers have been able to support the differentiation of dementia subtypes [75], which typically requires extensive and potentially invasive testing in clinical practice [76]. Digital technologies can also be used for monitoring disease progression, such as the electronic pillbox included in this review [31], which could be used to understand the best point to intervene with the care needs of individuals showing signs of progressing cognitive decline. However, a key barrier to their use is the lack of standardization and validation, which has been observed in previous reviews assessing digitally measured activities of daily living in cohorts with MCI and Parkinson disease and highlights a research gap [29,73]. Progress has been made in reporting some BADL modalities, such as the Mobilise-D framework, which describes the steps necessary to introduce clinically meaningful, valid, and reliable tools to facilitate remote visits and the monitoring of mobility outcomes [77]. The digital assessment of IADL changes in people with MCI is still at an early stage. This review has summarized the available literature on digital IADL assessment in people with MCI and highlighted areas of future research, which could help to progress this field.

### Strengths and Limitations of This Study

This systematic review has multiple strengths, including a comprehensive search methodology and independent screening of titles, abstracts, and full-text articles by 3 reviewers. Furthermore, a selected method was used to identify IADL domains through consensus to aid synthesis. Because of the heterogeneity in the metrics reported in this review, a meta-analysis was not appropriate; however, this would be useful in future research once metrics have been standardized. In addition, only articles written in English were considered for inclusion, which may have led to the exclusion of relevant research published in other languages.

### Conclusions

To conclude, this review found ambient technology (eg, motion sensors) to be the most common IADL assessment tool, whereas metrics related to *everyday technology use* were most prevalent in populations with MCI. The lack of standardization in the metrics used to assess IADL-related behaviors highlights the need for core metrics to be identified through patient and public involvement, based on clinically meaningful outcomes, to inform the selection of appropriate validated technologies for use in future research. Inconsistent findings and limited longitudinal assessment restrict our understanding; therefore, future research should focus on assessing relevant IADL-related behaviors using a range of available digital technologies, some of which may not yet be used in this space, and increasing the duration of monitoring to observe changes over time.

## Appendix

Multimedia Appendix 1 Search strategy and key terms for databases.

Multimedia Appendix 2 Quality assessment of all included studies.

Multimedia Appendix 3 Key findings related to the instrumental activities of daily living digital endpoints in mild cognitive impairment for all included studies.

Multimedia Appendix 4 Table of definitions and prevalence of all digital instrumental activities of daily living–related behavior metrics used in the included studies.

## Abbreviations

BADL: basic activity of daily living
CDR: clinical dementia rating
EDoN: Early Detection of Neurodegenerative Diseases
IADL: instrumental activity of daily living
MCI: mild cognitive impairment
MMSE: Mini-Mental State Examination
PIR: passive infrared
PRISMA: Preferred Reporting Items for Systematic Reviews and Meta-Analyses
RADAR-AD: Remote Assessment of Disease and Relapse–Alzheimer’s Disease
SCD: subjective cognitive decline
SPHERE: Sensor Platform for Healthcare in a Residential Environment
