# Supplementary material for: Digital Endpoints for Assessing Instrumental Activities of Daily Living in Mild Cognitive Impairment: Systematic Review
Source: J Med Internet Res. 2023 Jul 25;25:e45658. doi: 10.2196/45658 (PMC10410386; doi:10.2196/45658)
Supplement: Multimedia Appendix 1 [file jmir_v25i1e45658_app1.pdf]

Search Strategy

Search Strategy

**Category**	**String**	**Notes**
**Cognitive Impairment**	"cognitiv* impair*" or "memory impair*" or "cognitive decline*" or "cognitive dysfunction*" or "mental deterioration*" or "mild neurocognitive disorder*" or "preclinical AD" or "preclinical alzheimer* diseas*" or "preclinical dementia" or "cognitive deficit*" or MCI or aMCI or nMCI or mMCI or MCIa or MCD	
**Functional Independence**	"functional status" or "functional dependence" or "functional independence" or "independent living" or "activit* of daily living" or "daily living activit*" or "daily activit*" or ADL or ADLs or IADL or IADLs or "everyday functioning" or "functional assessment" or "functional abilit*" or "functional disabilit*" or "functional deficit*" or "functional capabilit*"	
**Digital**	digital or tech* or biotech* or sens* or wearabl* or device* or "fitness tracker*" or "mobile application*" or mobile* or app or apps or application* or telemedicine* or telehealth or mhealth or ehealth or smartphon* or smartwatch* or smart-home* or "smart home*" or smarthome* or "activity recognition" or "geographical positioning system" or "gps" or on-body or "on body" or biosens* or motion-track* or actigraphy or accelerometer* or gyroscope or "remote measur* tech*" or RMT or "digital biomark*" or "unobtrusive sens*" or "home monitoring" or "smart assessment" or "ambient sens*" or "inertial measurement unit*" or "home based sens*" or "home-based sens*" or "passive monitor*" or "continuous monitor*" or freeliving or "real world" or "real-world" or "in-home monitor*" or "in home monitor*"	
**Time Frame**	2004 - 2022	

("cognitiv* impair*" or "memory impair*" or "cognitive decline*" or "cognitive dysfunction*" or "mental deterioration*" or "mild neurocognitive disorder*" or "preclinical AD" or "preclinical alzheimer* diseas*" or "preclinical dementia" or "cognitive deficit*" or MCI or aMCI or nMCI or mMCI or MCIa or MCD) AND ("functional status" or "functional dependence" or "functional independence" or "independent living" or "activit* of daily living" or "daily living activit*" or "daily activit*" or ADL or ADLs or IADL or IADLs or "everyday functioning" or "functional assessment" or "functional abilit*" or "functional disabilit*" or "functional deficit*" or "functional capabilit*") AND (digital or tech* or biotech* or sens* or wearabl* or device* or "fitness tracker*" or "mobile application*" or mobile* or app or apps or application* or telemedicine* or telehealth or mhealth or ehealth or smartphon* or smartwatch* or smart-home* or "smart home*" or smarthome* or "activity recognition" or "geographical positioning system" or "gps" or on-body or "on body" or biosens* or motion-track* or actigraphy or accelerometer* or gyroscope or "remote measur* tech*" or RMT or "digital biomark*" or "unobtrusive sens*" or "home monitoring" or "smart assessment" or "ambient sens*" or "inertial measurement unit*" or "home based sens*" or "home-based sens*" or

"passive monitor*" or "continuous monitor*" or freeliving or "real world" or "real-world" or "in-home monitor*" or "in home monitor*")
