# Supplementary material for: Digital Endpoints for Assessing Instrumental Activities of Daily Living in Mild Cognitive Impairment: Systematic Review
Source: J Med Internet Res. 2023 Jul 25;25:e45658. doi: 10.2196/45658 (PMC10410386; doi:10.2196/45658)
Supplement: Multimedia Appendix 2 [file jmir_v25i1e45658_app2.docx]

Table 1. Quality Assessment for all included studies.

| Study | Was the research question or objective in this paper clearly stated? | Was the study population clearly specified and defined? | Were withdrawals reported and explained? | Were inclusion and exclusion criteria for participants defined and determined prior to the study onset? | Was a sample size justification, power description, or variance and effect estimates provided? | Were the outcome measures (dependent variables) clearly defined, valid, reliable, and implemented consistently across all study participants? | Were clinical diagnostic criteria and severity ratings for dementia reported and adhered to? | Were key potential confounding variables measured and adjusted statistically for their impact on the outcome(s)? | Quality Assessment: Reviewer 1 (LL) | Quality Assessment: Reviewer 2 (SW/RK) | Average Score |
| --- | --- | --- | --- | --- | --- | --- | --- | --- | --- | --- | --- |
| Bernstein et al. (2021) | Yes | Yes | No | Yes | Yes/No | Yes | Yes | No/Yes | Mediocre (6) | Mediocre (6) | Mediocre (6) |
| Dodge et al. (2015) | Yes | Yes | No | Yes | Yes | Yes | Yes | No | Mediocre (6) | Good (7) | Mediocre (6.5) |
| Dorociak et al. (2021) | Yes | Yes | No | Yes | No | Yes | Yes | Yes | Mediocre (6) | Mediocre (6) | Mediocre (6) |
| Hayes et al. (2008) | Yes | Yes | No | Yes/No | No | Yes | Yes | No/Yes | Mediocre (5) | Mediocre (5) | Mediocre (5) |
| Kaye et al. (2014) | Yes | Yes | Yes | Yes | No | Yes | Yes | Yes | Good (7) | Good (7) | Good (7) |
| Leese et al. (2021) | Yes | Yes | Yes/No | Yes | No | Yes | Yes | Yes | Good (7) | Mediocre (6) | Mediocre (6.5) |
| Liddle et al. (2021) | Yes | Yes | Yes/No | Yes/No | No/Yes | Yes | Yes/No | Yes | Good (7) | Mediocre (5) | Mediocre (6) |
| Petersen et al. (2015) | Yes | Yes | No/Yes | Yes | No | Yes | Yes | Yes | Mediocre (6) | Good (7) | Mediocre (6.5) |
| Rawtaer et al. (2020) | Yes | Yes | Yes | Yes | Yes | Yes/No | Yes | No | Good (7) | Mediocre (6) | Mediocre (6.5) |
| Seeyle et al. (2015) | Yes | Yes | No | Yes | No | Yes | Yes | Yes | Mediocre (6) | Mediocre (6) | Mediocre (6) |
| Seeyle et al. (2017) | Yes | Yes | Yes | Yes | No | Yes | Yes | No/Yes | Mediocre (6) | Good (7) | Mediocre (6.5) |
| Seeyle et al. (2020) | Yes | Yes | Yes | Yes | No | Yes | Yes/No | No/Yes | Mediocre (6) | Mediocre (6) | Mediocre (6) |
| Stringer et al. (2022) | Yes | Yes | No | Yes | Yes/No | Yes | Yes/No | Yes | Good (7) | Mediocre (5) | Mediocre (6) |
| Wettstein et al. 2015) | Yes | Yes | No | Yes | No/Yes | Yes | Yes | Yes | Mediocre (6) | Good (7) | Mediocre (6.5) |
| Wu et al. (2021) | Yes | Yes | No | Yes | No | Yes | Yes | Yes | Mediocre (6) | Mediocre (6) | Mediocre (6) |
