# Supplementary material for: Digital Endpoints for Assessing Instrumental Activities of Daily Living in Mild Cognitive Impairment: Systematic Review
Source: J Med Internet Res. 2023 Jul 25;25:e45658. doi: 10.2196/45658 (PMC10410386; doi:10.2196/45658)
Supplement: Multimedia Appendix 3 [file jmir_v25i1e45658_app3.docx]

Table 3 Key Findings related to IADL Digital Endpoints in MCI for all studies.

| Study | Domain | Technology | Duration | Key findings | Comparison |
| --- | --- | --- | --- | --- | --- |
|  |  |  |  |  |  |
| Bernstein et al. (2021) | Everyday Technology Use | Worktime computer use monitoring software to track activity on the computer, to operate in the background during use | 3 Months | Computer Use Variables  Computer Use time (minutes)  MCI: 63.4±36.4 Controls: 105.9±88.9  d: 0.57  Computer Use Time Variability (minutes)  MCI: 49.2±26.5  Controls: 67.8±43.0  d: 0.49  Number of Sessions  MCI: 7.3±3.8  Controls: 11.6±7.6  d: 0.65  Time of First Session  MCI: 11:53am±2:34:52  Controls: 10:04am±2:37:11  d: 0.70  Time of Last Session  MCI: 4:14pm±2:22:43  Controls: 5:04pm±2:41:15  d: 0.32  % Days with at least One Session  MCI: 55.3%±34.7  Controls: 70.4%±33.1  d: 0.45  Application Use Variables  Email Use Time (minutes)  MCI: 46.8±114.4  Controls: 398.4±841.0  d:0.53  Game Use Time, minutes  MCI: 424.4±1748.0  Controls: 504.7±1404.3  d: 0.07  Browser Use Time, minutes  MCI: 2602.9±2757.6  Controls: 5078.2±5212.6  d: 0.60  Teleconferencing Use Time, minutes  MCI: 0.0±0.0  Controls: 3.2±15.2  d: 0.26  Finance Use Time, minutes  MCI: 42.1±116.4  Controls: 30.4±111.6  d: 0.08  Search Use Time, minutes  MCI: 160.4±288.0  Controls: 436.2±1324.2  d: 0.27  Word Processing Use Time, minutes  MCI: 60.0±242.1  Controls: 299.9±542.3  d: 0.54  Email Use, Days  MCI: 6.5±13.8  Controls: 20.7±34.3  d: 0.52  Game Use, Days  MCI: 4.0 ±12.9  Controls: 11.4±25.7  d: 0.33  Browser Use, Days  MCI: 48.4±30.4  Controls: 61.7±30.6  d: 0.51  Teleconferencing Use, Days  MCI: 0.0±0.0  Controls: 0.4±2.0  d: 0.26  Finance Use, Days  MCI: 1.5±3.3  Controls: 2.6±10.3  d: 0.14  Search Use, Days  MCI: 23.6±18.4  Controls: 36.5±27.5  d: 0.59  Word Processing Use, Days  MCI: 1.8±3.7  Controls: 15.5±22.3  d: 0.77 | Computer Use Variables  MCI spent less time using the computer daily (*p* <.05), had less computer sessions (*p* <.05), a later first computer use time (*p* <.05), and less variability in their use time per day (*p* =.06)  Application Use Variables  MCI spent less minutes using email (*p* <.05), web browsers (*p* <.05), and word processing (*p* <.05) than controls, and less total number of days using search tools (*p*<.05), and word processing (*p* <.01) |
| Dodge et al. (2015) | Everyday Technology Use | Mouse movement data to operate in the background during use | 3 months  Baseline: 1 week | Baseline Mean Daily Computer Usage, minutes  Incident MCI: 110.94±86.08  Controls: 73.91±51.81 | Computer usage not significant different at baseline. Data not collected after incident MCI occurred.  Only computer usage demonstrated a significant difference in trajectories between the two groups *p* = 0.01. Controls had less decline over time in weekly average minutes on computer than incident MCI subjects.  Those destined to develop MCI spent fewer minutes on their computer over time. |
| Dorociak et al. (2021) | Medication Management | Medtracker, 7-day electronic, wireless pillbox that tracks adherence by detecting opening/closing of each door. | Minimum 2 weeks of data, Mean: 2.3 years  Baseline: 30 days | Baseline Medication Adherence  MCI: 83±27  Controls: 85±24  Baseline mean pill-taking time of day, hours  MCI: 8:32am±0.8  Controls: 9:47am±4  Baseline variability in pill-taking time, hours  MCI: 2.6±1.9  Controls: 2.4±1.8  Incident MCI * monitoring time  Pill-taking Time of Day  β Coefficient: 0.46  *P*-value: <.001  Variability in pill-taking time (minutes)  β Coefficient: 4.0  *P*-value: .003  Number of days missed  β Coefficient: 0.002  *P*-value: .17 | No significant differences at first month in any metric.  Incident MCI individuals opened their pillboxes increasingly later in the day (by 19 minutes per month), *p* < .001  d incident MCI participants became significantly more variable in the time of their first pillbox opening each day (by four minutes per month) *p* = .003 |
| Hayes et al. (2008) | Activities Outside of the Home | Passive infrared pyroelectric motion sensors (MS16A, x10.com) placed in every room at locations expected to pick up the participant’s movements  Magnetic contact sensors (DS10A, x10.com) were placed on each door of the home to track visitors and absences from the home | 26 weeks | Outings per day  MCI: 0.96 ± 0.67  Controls: 2.03 ± 1.43  Time out of home (minutes/day)  MCI 62.0 ± 70.9  Controls: 198.0 ± 142.3  24-hour wavelet variance  MCI: 4.07 ± 0.14  Controls: 3.79± 0.23  Daily Activity Estimate MCI: 0.79±0.14 Controls: 0.70±0.15 | Day-to-day pattern of activity of subjects in the MCI group was more variable than controls *p* <0.008. |
| Kaye et al. (2014) | Everyday Technology Use | Mouse movement data to operate in background during use  PIR motion sensors in each room in locations expected to pick up the participant’s movements  Contact sensor on front door to track visitors and absences from the home | Mean: 36 Months Baseline: 1 month | Baseline days with computer use  MCI: 17.7±8.0  Controls: 19.8±8.9  Baseline daily mean use  MCI: 1.5±1.7  Controls: 1.5±1.1  Baseline COV of use  MCI: 129.9±66.1  Controls: 112.2±65.1  MCI x Duration  days on computer per month:  coefficient: -0.119  *P*-value: .01  daily mean use per month  coefficient: -0.008  *P*-value: <.01  COV of use per month  coefficient: 0.010  *P*-value: .002 | MCI used computer for less days per month than controls over time, *p* = .01.  MCI had decrease in mean daily use over time, ~1% greater decrease per month, *p* <.01.  MCI had increasing variability in use over time compared to controls, *p* <.002. |
| Leese et al. (2021) | Everyday Technology Use  Activities Outside of the Home | Worktime computer use monitoring software to track activity on the computer, to operate in the background during use  Passive driving sensor, Automatic Pro Sensor, to monitor multiple driving metrics, plugged into vehicle data-port | 5 Months | Daily Distance (estimate)  MCI vs Controls: 0.13 95% CI: −0.07, 0.33  Daily Number of Trips (estimate)  MCI vs Controls: 0.07 95% CI: −0.07, 0.20  Daily Computer use time minutes (estimate)  MCI vs Controls: −0.10  95% CI: −0.67, 0.46  Daily Highway driving ratio  MCI vs Controls: 1.06  95% CI: 0.44, 2.51  Daily night time driving ratio  MCI vs Controls: 0.93  95% CI: 0.50, 1.73  Daily driving time (baseline) (mins)  MCI: 65.2 (19.7)  Controls: 61.2 (22.2)  Daily computer usage (baseline) (mins)  MCI: 40.5 (29.6)  Controls: 64.2 (52.7) | No significant differences between groups. |
| Liddle et al. (2021) | Activities Outside of the Home;  Orientation | Smartphone app to passively record GPS location outdoors, worn on the body  Low-energy Bluetooth beacons (3-5) distributed around participants’ homes, one per key room to measure motion inside the home. | 1 Week | Percentage of time at home Median (IQR) range  MCI: 92.14% (10.52) 65.03–99.72  Dementia: 86.59% 84.62–93.95  Average maximum distance from home (km) Median (IQR) range  MCI: 1.23 (1.79) 0.16–5.68  Dementia: 2.59 1.26–4.12  Trips away from home in a week Median (IQR) range  MCI: 5.00 (5.00) 0–16  Dementia: 7.00 5–12  Days in the week when left home Median (IQR) range  MCI: 3 (4) 0–7  Dementia: 5.00 4–7.00  Daily lifespace area (km^2^)  MCI: 1.75 (2.56) 0.11–10.47  Dementia: 1.43 0.62–5.10  Total lifespace area (km^2^)  MCI: 5.72 (13.02) 0.10–97.88  Dementia: 12.25 6.87–54.66  Lifespace score  MCI: 8.00 (4.00) 4.00–11.00  Dementia: 8.00 8.00–10.00  Indoor lifespace  MCI: 9.72 (19.88) 0–25.66  Dementia: 15.28 6.35–19.95 | No significant relationships between lifespace metrics and cognitive status.  Changes from baseline cognitive status: Wave 4  15 MCI  3 Dementia (unspecified) |
| Petersen et al. (2015) | Activities Outside of the Home | Pyroelectric infrared motion sensors (MS16A, x10.com) in each room in locations expected to pick up the participant’s movements  Contact sensors (DA10A, x10.com) on the refrigerator and doors to the home to track use, as well as visitors and absences from the home | Mean 227 days |  | MCI spent an average of 1.67 hours more inside the home than controls  And were 12% less likely to leave the home at all on any given day, *p* <0.001 |
| Rawtaer et al. (2020) | Medication Management;  Household and Personal Management;  Activities Outside of the Home | Passive infrared (PIR) motion sensors, one per room in locations expected to pick up the participant’s movements  Contact sensor, on door to outside to track visitors and absences from the home  Proximity beacon tags attached to keys and wallet to track their location  sensor-equipped medication box that tracks adherence by detecting opening/closing of each door, located in home .  Wearable activity band, Microsoft band, worn on wrist | 2 Months | Number of outings daily  MCI: 1±1  Controls: 1±1  Time away from home daily (min)  MCI: 267±132  Controls: 300±153  Frequency of forgetting medication/month  MCI: 30±28  Controls: 28±13  Frequency of forgetting keys per month  MCI: 17±13  Controls:21±16  Frequency of forgetting wallet per month  MCI: 24±22  Controls: 24±17 | No significant differences between groups. |
| Seeyle et al. (2015) | Everyday Technology Use | Mouse movement data, to operate in background during computer use | 1 week | Median delta  MCI: 36.5±14.6  Controls: 50.6±23.0  IQR delta  MCI: 112.0±50.4  Controls: 138.2±50.5  Median D  MCI: 42.3±19.3  Controls: 56.5±24.6  IQR D  MCI: 123.7±55.9  Controls: 150.5±54.7  Median T  MCI: 199.9±55.5  Controls: 237.2±72.2  IQR T  MCI: 325.7±143.7  Controls: 332.6±122.2  Median K  MCI: 0.87±0.03  Controls: 0.88±0.02  IQR K  MCI: 0.15±0.02  Controls: 0.14±0.02  Median idle  MCI: 346.6±104.6  Controls: 308.7±61.4  IQR idle  MCI: 1249.9±942.6  Controls: 832.0±424.6  Number of mouse movements contributed  MCI: 1497±1684 *p* <.01  Controls: 7871±9679  Number of computer sessions contributed MCI: 4.9±4.2  Controls: 9.5±11.2 | MCI had shorter movements (delta, D) than controls, *p* <.05  MCI took less time to make individual movements, *p* <.05  MCI had larger and more variable number of curved/looped movements (less direct) (IQR_K; (*p* <.05)  MCI had larger and mor variable length of pauses between movements (IQR_Idle; *p* < .05.  MCI made fewer total movements than controls , *p* <.01 |
| Seeyle et al. (2017) | Activities Outside of the Home | Passive driving sensor, Automatic and Automatic mobile app located in-vehicle | 206 ± 36 days | Mean # of trips per day  MCI: 4.7±1.4  Controls: 4.1±0.9  Day-to-day variability in # of trips  MCI: 2.3±0.8  Controls: 2.1±0.5  Mean distance driven per day (miles)  MCI: 14±11  Controls: 22±13  Day-to-day variability in distance driven  MCI: 13±12  Controls: 31±17  Mean time driven per day (h)  MCI: 0.8±0.4  Controls: 0.9±0.4  Day-to-day variability in time driven  MCI: 0.5±0.2  Controls: 0.8±0.3  Mean first clock start time of driving per day  MCI: 10.36±1.4  Controls: 11.18±1.2  Day-to-day variability in first start time (h)  MCI: 2.7±1.1  Controls: 2.8±0.6  Mean last clock start time of driving per day  MCI: 15.9±1.9  Controls: 15.1±1.4  Day-to-day variability in last start time (h)  MCI: 3.2±0.8  Controls: 3.2±0.6  Mean # of days monitored  MCI: 201±33  Controls: 208±38  % of days at least one trip was taken out of all days monitored  MCI: 60%  Controls: 49%  % of driving days with ≥20 miles driven  MCI: 21%  Controls: 27%  Mean time of highway driving per day (s)  MCI: 172±288  Controls: 543±533  Mean time of night-time driving per day (s)  MCI: 337±571  Controls: 191±194  Mean # left turns per day  MCI: 9.2±3  Controls: 7.9±3.3  Mean # right turns per day  MCI: 10.9±3  Controls: 9.6±4.1  Mean time driving over 70 mph from day (s)  MCI: 21±53  Controls: 44±62  Mean # of hard breaks per day  MCI: 0.7±0.6  Controls: 1.3±0.9  Mean # of hard accelerations per day  MCI: 0.7±0.7  Controls: 0.8±0.9  MCI*time  Daily Total Driving Distance  Coefficient:-.001  *P*-value: 0.24  Daily Total Driving Time  Coefficient:-.001  *P*-value: 0.08 | MCI less variable in daily driving distance (*p* =.01) and time (*p* <.01)  MCI drove less miles (*p* =.06), and spent less time driving on highway than controls (*p* <.01).  No change over time (exploratory longitudinal analysis) |
| Seeyle et al. (2020) | Medication Management;  Everyday Technology Use | TimerCap iSort 7-day pillbox, tracks adherence by detecting opening/closing of each door, located in home.  Worktime computer monitoring software, to track activity on the computer, operating in the background during use | 7 Months | Total device repair visits  MCI: 3.1±1.9  Controls: 1.8±1.2  Participants requiring >1 pillbox visit  MCI: 6 (40%)  Controls: 1 (7%)  Participants requiring >1 worktime visit  MCI: 6 (40%)  Controls: 5 (33%) | No significant differences |
| Stringer et al. (2022) | Everyday Technology Use | Computer monitoring system (SAMS) recording mouse clicks and keystrokes during use | 7 -9 Months | Daily Computer Use (minutes)  MCI: 43.95±66.46  SCD: 87.14±112.01  Mouse click frequency per minute  MCI: 7.47±5.94  SCD: 8.08±6.73  Keystroke speed (s)  MCI: 2.05±0.64  SCD: 2.92±0.71  MCI*time (Adjusted)  Daily computer use  β Coefficient: -.032  *P*-value: .417  Mouse click frequency  β Coefficient: -.002  *P*-value: .437  Keystroke Speed  β Coefficient: .000  *P*-value: .109 | MCI spent significantly less time on the computer (*p* = .026) and had slower keystroke speed (*p* < .001) compared to individuals with SCD.  No change over time in computer use variables during study period. |
| Wettstein et al. (2015) | Activities Outside of the Home | Portable GPS receiver with GSM modem and monitoring unit, worn in belly pouch/shoulder bag | 4 weeks | Time out-of-home (hours)  MCI: 4.3±2.3; 0.3-9.9  AD: 2.7±1.5; 0-7.3  Controls: 4.2±2.3; 0.7-12.8  Number of Nodes Visited:  MCI: 4.6±1.4; 1.4-8.2  AD: 3.6±1.2; 1.1-6.6  Controls: 4.7±1.3; 1.9-9.4 | AD spent significantly less time out-of-home than MCI and controls, *p* <.01, and visited less nodes, *p* <.001. |
| Wu et al. (2021) | Activities Outside of the Home;  Orientation | Passive infrared (PIR) motion sensors were fixed on the wall in four major rooms [bathroom(s); bedroom(s); kitchen(s); living room(s)] in locations expected to pick up the participant’s movements  Contact sensors were also fixed on the front door (entrance) to track visitors and absences from the home | M 206 days ±127 | Indoor Mobility Frequency ICC (95% CI): 0.91(0.88-0.94)  MCID: 18(SEM)  MCI: 118.7±53.6 baseline  Controls: 111.1±63.1 baseline  Indoor Mobility Stability ICC (95% CI): 0.59 (0.48-0.7)  MCID: 0.09(SEM)  MCI: 0.3±0.1 Baseline  Controls: 0.4±0.1 Baseline | Lower indoor mobility stability (higher day-to-day variability per week) was associated with MCI status, *p* =.01. Significant after adjusting for age, gender, race, education, pain, low mood, no. of rooms, hours of daylight and time out-of-home, *p* =.03. |

Abbreviations: MCI = Mild Cognitive Impairment, SCD = Subjective Cognitive Decline, ICC = Intra-correlation coefficient, AD = Alzheimer’s Disease, COV = Coefficient of variance, d = Cohen’s d, CI = Confidence interval, MCID = Minimal clinically important difference, SEM = Standard error of measurements, SAMS = Software Architecture for Mental Health Self-Management, GPS = Geographical positioning system, GSM = Global Systems for Mobile communications.
