# Supplementary material for: Digital Endpoints for Assessing Instrumental Activities of Daily Living in Mild Cognitive Impairment: Systematic Review
Source: J Med Internet Res. 2023 Jul 25;25:e45658. doi: 10.2196/45658 (PMC10410386; doi:10.2196/45658)
Supplement: Multimedia Appendix 4 [file jmir_v25i1e45658_app4.docx]

Table of definitions and prevalence of all digital IADL related behaviour metrics used in included studies.

| Metric | Percentage (%) of papers assessing metric (n) | Description |
| --- | --- | --- |
|  |  |  |
| **Everyday Technology Use** |  |  |
| Computer use time | 40% (6)* | Daily time, in minutes, spent using the computer |
| Computer use time variability | 6.67% (1) | Day-to-day variation in minutes spent using the computer |
| Number of sessions | 20% (3)* | Number of daily uses of the computer |
| Time of first session | 13.33% (2)* | Time of day using the computer for the first time |
| Time of last session | 13.33% (2)* | Time of day using the computer for the last time |
| % Days with at least one session | 6.67% (1) | Percentage of days with at least one session using the computer |
| Days on computer per month | 6.67% (1) | Average number of days spent using the computer per month |
| Coefficient of variation of use | 6.67% (1) | A measure of the variability or consistency in day-to-day use per month |
| Email use time, minutes | 6.67% (1) | The total amount of time, in minutes, that each participant used email over the course of the study period |
| Email use, days | 6.67% (1) | The total number of days in which each participant used at least one email application across the study period |
| Game use time, minutes | 6.67% (1) | The total amount of time, in minutes, that each participant used computer games over the course of the study period |
| Game use, days | 6.67% (1) | The total number of days in which each participant used at least one computer game application across the study period |
| Browser use time, minutes | 13.33% (2)* | The total amount of time, in minutes, that each participant used the computer browser over the course of the study period |
| Browser use, days | 6.67% (1) | The total number of days in which each participant used at least one browser application across the study period |
| Teleconferencing use time, minutes | 6.67% (1) | The total amount of time, in minutes, that each participant used teleconferencing over the course of the study period |
| Teleconferencing use, days | 6.67% (1) | The total number of days in which each participant used at least one teleconferencing across the study period |
| Finance use time, minutes | 6.67% (1) | The total amount of time, in minutes, that each participant used financial applications over the course of the study period |
| Finance use, days | 6.67% (1) | The total number of days in which each participant used at least one financial application across the study period |
| Search use time, minutes | 6.67% (1) | The total amount of time, in minutes, that each participant used the search tool over the course of the study period |
| Search use, days | 6.67% (1) | The total number of days in which each participant used at least one search tool application across the study period |
| Word processing use time, minutes | 13.33% (2)* | The total amount of time, in minutes, that each participant used word processing over the course of the study period |
| Word processing use, days | 6.67% (1) | The total number of days in which each participant used at least word processing application across the study period |
| Median delta | 6.67% (1) | Median straight-line distance travelled by the mouse, between starting and end points, in counts. |
| IQR delta | 6.67% (1) | Interquartile range of the straight-line distance travelled by the mouse, between starting and end points, in counts. |
| Median D | 6.67% (1) | Median total distance travelled by the mouse in counts |
| IQR D | 6.67% (1) | Interquartile range of the total distance travelled by the mouse in counts |
| Median T | 6.67% (1) | Median time taken to make a mouse movement in milliseconds |
| IQR T | 6.67% (1) | Interquartile range of the time taken to make a mouse movement in milliseconds |
| Median K | 6.67% (1) | Median mouse movement curvature, ranging from 0 to 1 (0 = looped, 1 = straight line) |
| IQR K | 6.67% (1) | Interquartile range of mouse movement curvature |
| Median idle | 6.67% (1) | Median time spent idling or pausing between successive mouse movements in milliseconds |
| IQR idle | 6.67% (1) | Interquartile range of time spent idling or pausing between successive mouse movements in milliseconds |
| Number of mouse movements contributed | 6.67% (1) | Total number of mouse movements per session |
| Number of computer sessions contributed | 6.67% (1) | Total number of computer sessions across the study duration |
| Mouse click frequency | 6.67% (1) | Total mouse clicks (left and right) per day divided by the total duration of computer-use per day |
| Daily keystroke speed | 6.67% (1) | The total number of keystrokes in bursts (a series of at least five consecutive keystrokes with a pause no longer than 1.957s between keys) per day divided by the total duration of bursts per day |
|  |  |  |
| **Medication Management** |  |  |
| Medication adherence | 13.33% (2)* | Number of days with door opening divided by total days multiplied by 100 |
| Pill-taking clock time | 13.33% (2)* | Average time of day door opened in minutes from midnight |
| Pill-taking clock time variability | 6.67% (1) | Standard deviation of first time door opened each day, in hours |
| Frequency of forgetting medication per month | 6.67% (1) | The number of times a participant forgot to take their medication at the prescribed time per month |
|  |  |  |
| **Household and Personal Management** |  |  |
| Frequency of forgetting keys per month | 6.67% (1) | The number of times a participant forgot to take their keys outside of the home each month |
| Frequency of forgetting wallet per month | 6.67% (1) | The number of times a participant forgot to take their wallet outside of the home each month |
|  |  |  |
| **Activities Outside of the Home** |  |  |
| Daily distance (meters) | 6.67% (1) | Furthest distance travelled from home in a vehicle per day, in metres |
| Mean # of trips per day | 13.33% (2) | Number of trips in the car per day |
| Day-to-day variability in # of trips | 6.67% (1) | Standard deviation of the mean number of trips per day |
| Mean distance driven per day (miles) | 6.67% (1) | The average number of miles driven per day |
| Day-to-day variability in distance driven | 6.67% (1) | Standard deviation of the average number of miles driven per day |
| Mean time driven per day (min) | 13.33% (2) | The average time spent driving per day, in minutes |
| Day-to-day variability in time driven | 6.67% (1) | Standard deviation of the average number of minutes spent driving per day |
| Mean first clock start time of driving per day | 6.67% (1) | The average time of day for the first driving trip, per day |
| Day-to-day variability in first start time (h) | 6.67% (1) | Standard deviation of the average time of day for the first driving trip, in hours |
| Mean last clock start time of driving per day | 6.67% (1) | The average time of day for the last driving trip, per day |
| Day-to-day variability in last start time (h) | 6.67% (1) | Standard deviation of the average time of day for the last driving trip, in hours |
| Mean # of days monitored | 6.67% (1) | The average number of days in which participants’ driving was monitored during the study |
| % of days at least one trip was taken out of all days monitored | 6.67% (1) | The number of days with at least one instance of driving out of the total number of monitored days, as a percentage |
| % of driving days with ≥20 miles driven | 6.67% (1) | The number of days participants drove for 20 miles or further, out of the total monitored days |
| Mean time of highway driving per day (s) | 13.33% (2) | The average number of seconds spent driving on a highway, per day |
| Mean time of night-time driving per day (s) | 13.33% (2) | The average number of seconds spent driving after dusk, per day |
| Mean # left turns per day | 6.67% (1) | The average number of left turns whilst driving per day |
| Mean # right turns per day | 6.67% (1) | The average number of right turns whilst driving per day |
| Mean time driving over 70 mph from day (s) | 6.67% (1) | The average number of seconds spend driving at a speed higher than 70mph per day |
| Mean # of hard breaks per day | 6.67% (1) | The average number of times participants applied more force than normal to decrease the speed of their vehicle, per day |
| Mean # of hard accelerations per day s17 | 6.67% (1) | The average number of times participants applied more force than normal to increase the speed of their vehicle, per day |
| Mean number of outings per day | 13.33% (2) | Number of times per day that participants left the home |
| Trips away from home in a week | 6.67% (1) | Total number of times a participant remained 500 metres or further from the home for a time longer than 5 minutes before returning in a week |
| Time out-of-home | 40% (6)* | Number of minutes/hours spent outside of the home per day |
| Daily activity estimate | 13.33% (2)* | Number of sensor firings per minute based on the time the subject was in the home |
| Coefficient of variation of activity | 6.67% (1) | Ratio of standard deviation to the mean for daily activity |
| 24-hour wavelet analysis of activity variance | 6.67% (1) | A method of decomposing a time-varying signal at multiple resolutions used to examine differences in 24-hour activity variance over time |
| Percentage of time at home (%) | 6.67% (1) | A percentage of recorded time spent at home over a week |
| Average maximum distance from home | 6.67% (1) | The mean furthest distance travelled from home, in kilometres |
| Days in the week when left home | 6.67% (1) | Average number of days in which participants left their home |
| Number of nodes visited daily | 6.67% (1) | Average number of places visited per day |
|  |  |  |
| **Orientation** |  |  |
| Indoor mobility frequency | 6.67% (1) | Total number of room-to-room transitions per day |
| Indoor mobility stability | 6.67% (1) | The variance of number of room-to-room transitions within a week |
| Daily lifespace area | 6.67% (1) | The average geographical area travelled per day, in kilometres squared. |
| Indoor lifespace | 6.67% (1) | The average number of different regions in the home occupied per hour |
| Total lifespace area | 6.67% (1) | The overall geographical area travelled by the participant in kilometres squared. |
| Lifespace score | 6.67% (1) | An additional lifespace metric using combined percentage of time at home, number of trips away from home, maximum distance and lifespace area; scores ranging from 4 – 12 where higher scores indicate a larger lifespace. |

* denotes a metric where 1 or more studies described using, but did not report the metric results.
